# Editorial: Cell Biology, Physiology and Molecular Pharmacology of G Protein Coupled Receptors

**DOI:** 10.3389/fcell.2021.815291

**Published:** 2021-12-09

**Authors:** Manveen Kaur Gupta, Muheeb Beg, Sameer Mohammad

**Affiliations:** ^1^ Cardiovascular and Metabolic Sciences, Lerner Research Institute, Cleveland Clinic & Case Western Reserve University, Cleveland, OHIO, United States; ^2^ Department of Medical Biochemistry and Cell Biology, Institute of Biomedicine, Sahlgrenska Academy, University of Gothenburg, Gothenburg, Sweden; ^3^ Experimental Medicine, King Abdullah International Medical Research Center (KAIMRC), King Saud Bin Abdulaziz University for Health Sciences (KSAU-HS), Ministry of National Guard Health Affairs (NGHA), Riyadh, Saudi Arabia

**Keywords:** GPCR (G protein coupled receptor), adrenergic receptor (AR), GPCR drug discovery, GRK (G protein receptor kinase), Incretin – Based therapy

G protein-coupled receptors (GPCRs) are the largest family of cell-surface proteins. They are characterized by seven transmembrane domains; a cytoplasmic C-terminus, and an extracellular N-terminal domain. GPCRs respond to a wide variety of signaling molecules and signal through the trimeric G-protein complex. GPCRs are expressed essentially on all cells, facilitating cellular responses to external stimuli and are involved in nearly every biological process (Pierce et al., 2002; Strange, 2008; Hanyaloglu and Grammatopoulos, 2017; Pavlos and Friedman, 2017; Wang et al., 2018). There are ∼800 members of the GPCR family, of them more than 400 are sensory receptors (olfactory, vision, and taste receptors (Alexander et al., 2019). The remaining ∼350 are non-sensory receptors and are activated by physical ligands, which include peptide hormones, large polypeptides, amino acids and small metabolites, free fatty acids and many others (Okuno et al., 2006; Mohammad, 2015; Wolf and Grünewald, 2015; Husted et al., 2017; Al Mahri et al., 2020; Davenport et al., 2020). Based on sequence homology, GPCRs are divided into different families: Class A (rhodopsin), Class B (secretin, adhesion), Class C (Glutamate), and frizzled receptors (Alexander et al., 2019). Most of these receptors have a known physical ligand, which activates the receptor to elicit the signaling cascade and the downstream effect on physiological function. GPCRs are substantially involved in human pathophysiology and are pharmacologically tractable, making them the most intensely studied drug targets. Nearly one-third of all drugs approved by the US Food and Drug administration involve GPCR target sites. In addition, several GPCR based drugs are currently undergoing clinical trials (Nieto Gutierrez and McDonald, 2018; Sriram and Insel, 2018). However, only about 100 GPCRs have been extensively studied and successfully targeted while the functional relevance of a significant number of GPCRs remains to be studied. Therefore, intense efforts are on to expansively study the rest of the members of the GPCR family to unearth additional therapeutic possibilities.

This research topic comprises of research papers and review articles on diverse GPCRs and their regulators. The collection of articles highlights the role of GPCRs in physiology, pathophysiology, and explores the possibility of exploiting their therapeutic potential. Perez (2021) reviews current developments on the role of α1-Adrenergic Receptors (α1-ARs) in cognition, cardioprotection, and metabolism. α1-ARs belong to the family of adrenergic receptors and have been extensively studied for their role in blood pressure regulation, cardiac hypertrophy, and muscle contraction. α1-ARs are also highly expressed in the cognitive centers of the brain and their activation has a profound effect on learning and memory function. The review highlights the potential of α1A-AR agonists or positive allosteric modulators to treat Alzheimer’s disease and to protect the heart at the same time.

Lin et al. (2021) in their paper demonstrate the role of Adenosine Receptor (AdoR) signaling and its downstream targets in *Drosophila*. They demonstrate that AdoR signaling represents an important pathway in response to stress conditions such as cytotoxicity, oxidative damage, and thermal stress in *Drosophila*. The paper provides important insights into the molecular mechanism of Ado regulation of stress response that could help understand how Ado signaling affects disease pathogenesis.

An interesting paper by Chou et al. (2021) shows how G-Protein-Coupled Estrogen Receptor-1 (GPER-1) positively regulates chondrocyte proliferation at the growth plate during early puberty and contributes to the longitudinal growth of long bones. GPER-1 is widely expressed in both mouse and human tissues including bone and cartilage. Interestingly, during pubertal progression, the expression of GPER-1 shows a significant decrease suggesting its involvement in the modulation of pubertal bone growth. Previous studies have investigated the expression of GPER-1 in bone and cartilage but the functional significance of GPER-1 in bone growth remains unclear. Using chondrocyte-specific GPER-1 knockdown mice, the authors demonstrate that GPER-1 positively regulates chondrocyte proliferation at the growth plate during early puberty and contributes to the longitudinal growth of long bones.

In a comprehensive review (Morrow et al., 2021), critically assess the role of incretin receptors, GIP receptor (GIPR), GLP-1 receptor (GLP-1R), and GLP-2 receptor (GLP-2R) in intestinal physiology. Incretin hormones (GIP and GLP-1) are gut peptides that are released from intestinal L- and K-cells respectively in response to food intake. Once released into the bloodstream, they bind to incretin receptors (GIPR. GLP-1R and GLP-2R) in pancreatic β-cells and enhance insulin releases in a glucose-dependent manner. In subjects with type 2 diabetes, this incretin effect is diminished or no longer present. Therefore, incretin-based therapies have been successfully used in diabetic patients. The authors in this paper highlight the biology and paracrine roles of GLP-1, GIP, and GLP-2 in integrating the response to food intake with the maintenance of the structure and function of the gut as it relates to nutrient absorption. A thought-provoking paper included in this research topic is by Kizilkaya et al. (2021) on incretin receptor GIPR. The authors functionally characterized two missense GIPR variants, R190Q (rs139215588) and E288G (rs143430880) that are associated with lower body mass index (BMI). The authors show that two naturally occurring rare GIPR variants, R190Q and E288G (rs139215588 and rs143430880, respectively), result in impaired GIPR function at the molecular level which in turn seems to impact human physiology and pathophysiology regarding adiposity, bone health, and cardiovascular system. These results indicate that GIPR antagonists could protect from diet-induced obesity and improve glycemic and insulinotropic effects, which is in contrast to other studies that have shown the beneficial effect of GIPR agonists on adipose metabolism. Previous studies show that how a single amino acid substitution in the GIPR receptor (E354Q) leads to enhanced agonist induced desensitization that impairs the ability of the GIP to control adipose insulin sensitivity (Mohammad et al., 2014). The data from these studies add to the interesting debate whether GIPR activation or GIPR inhibition is the right strategy to treat metabolic abnormalities associated with Type-2 diabetes.

It is widely documented that GPCR signaling involves cross-regulation of many pathways including cross-talks between different GPCRs as well as with other signaling pathways. Besides the acute signaling, GPCR, in direct or through crosstalk, also regulate the development of addictive diseases. In this area (Maccioni et al., 2021), in their paper demonstrate that treatment with non-sedative doses of the novel positive-allosteric modulator (PAM) of the GABA-B receptor, KK-92A ([(4-(cycloheptylamino)-5-(4-(trifluoromethyl)phenyl)pyrimidin-2-yl)methanol]) potently and effectively suppressed operant oral alcohol self-administration and cue-induced reinstatement of alcohol-seeking in alcohol-preferring Sp rats. KK-92A has high potency and selectivity for GABA-B. Besides, KK-92A has high bioavailability in the brain and a remarkable *in vivo* efficacy. The data from this study add to the earlier experimental data on the ability of KK-92A to reduce nicotine self-administration and cue-induced reinstatement of nicotine seeking in rats and therefore, broadening the anti-addictive profile of KK-92A.

Matthees et al. (2021) elucidate the role of GPCR Kinases (GRKs) in the regulation of GPCR signaling. GRKs and β-arrestins interact with activated GPCRs and regulate their intracellular trafficking. The authors discuss how the expression levels of GRKs, arrestins, and GPCRs play a crucial role in the development of pathological conditions. They analyzed expression data for GRKs and β-arrestins in 61 tissues annotated in the Human Protein Atlas and presented their analysis in the context of pathophysiological dysregulation of the GPCR/GRK/β-arrestin system. This tissue-specific point of view might be the key to unraveling the individual impact of different GRK isoforms on GPCR regulation.

The review by Tian et al. (2020) highlights recent progress regarding the critical components of the JAK2-STAT5 pathway and its crosstalk with G-protein coupled receptor (GPCR) signaling. Hormones are crucial for ductal morphogenesis in the mammary gland. During puberty, estrogen, growth hormone (GH), and prolactin are required for the development of the mammary gland. GH and prolactin regulate mammary gland function through the phosphorylation of Janus kinase 2 (JAK2) and activation of its downstream regulator signal transducers and activators of transcription 5 (STAT5). The authors evaluate recent data to demonstrate that GPCR activation has a profound impact on the JAK2-STAT5 signaling pathway.

Finally (Li et al., 2021), describe Identification and Functional Analysis of G Protein-Coupled Receptors in steroid hormone, 20-Hydroxyecdysone (20E) signaling from the Helicoverpa armigera Genome. The authors show that 20-hydroxyecdysone signals through multiple GPCRs including prolactin-releasing peptide receptor (PRRPR), smoothened (SMO), adipokinetic hormone receptor (AKHR), 5-hydroxytryptamine receptor (HTR), Frizzled 7 (FZD7), and tachykinin-like peptides receptor 86C (TKR86C) to regulate growth and development of Helicoverpa armigera.

## Conclusion

In conclusion, the research topic contains a fascinating collection of original research papers and review articles encompassing cell biology, physiology, and molecular pharmacology of G-protein coupled receptors. We hope that the data and information conveyed through this research topic will be beneficial to the scientific community in general and researchers in this exciting research area, in particular. We believe the research ideas presented will push more studies to further understand the physiological significance of GPCRs and unearth additional therapeutic possibilities.
